# Histone deacetylase inhibitors, glutamatergic drugs and deep brain stimulation rescue resistance to fear extinction in a genetic mouse model

**DOI:** 10.1186/2050-6511-13-S1-A42

**Published:** 2012-09-17

**Authors:** Nigel Whittle, Claudia Schmuckermair, Ozge Gunduz Cinar, Markus Hauschild, Francesco Ferraguti, Andrew Holmes, Nicolas Singewald

**Affiliations:** 1 Department of Pharmacology and Toxicology, Institute of Pharmacy and Center for Molecular Biosciences Innsbruck (CMBI), University of Innsbruck, 6020 Innsbruck, Austria; 2 Laboratory of Behavioral and Genomic Neuroscience, National Institute on Alcoholism and Alcohol Abuse, National Institutes of Health, Bethesda, MD 20852 and Center for Neuroscience and Regenerative Medicine at the Uniformed Services University of the Health Sciences, Bethesda, MD 20814, USA; 3 Department of Pharmacology, Innsbruck Medical University, 6020 Innsbruck, Austria

## Background

Impaired extinction of fear is a hallmark of a variety of disabling anxiety disorders including panic disorder, post-traumatic stress disorder, social anxiety disorder and specific phobias. Therapeutic interventions that reverse deficits in fear extinction represent a tractable approach to treating these disorders. We recently revealed that 129S1/SvImJ (129S1) mice are unable to extinguish learned fear responses following ‘normal’ fear conditioning, establishing these mice as a clinically relevant model to identify extinction-facilitating targets.

## Methods

129S1 mice were subjected to multi-trial ‘normal’ and ‘weak’ cued fear conditioning/extinction paradigms and novel treatment strategies to rescue deficient extinction were tested.

## Results

Results revealed that ‘weak’ fear conditioning permitted fear reduction during massed extinction training in 129S1 mice, but also revealed a specific deficiency in extinction memory consolidation/retrieval. Rescue of this impaired extinction consolidation/retrieval was achieved with D-cycloserine (*N*-methly-D-aspartate partial agonist) or MS-275 (histone deacetylase (HDAC) inhibitor), applied after extinction training. We next examined the ability of different drugs and non-pharmacological manipulations to rescue the extreme fear extinction deficit in 129S1 following ‘normal’ fear conditioning with the ultimate aim to produce low fear levels in extinction retrieval tests. Results showed that rescue of both impaired extinction acquisition and deficient extinction consolidation/retrieval was achieved with prior extinction training administration of valproic acid (a GABAergic enhancer and HDAC inhibitor) or AMN082 [metabotropic glutamate receptor 7 (mGlu7) agonist], while MS-275 or PEPA (AMPA receptor potentiator) failed to affect extinction acquisition in 129S1 mice. Lastly, deep brain stimulation (DBS) by applying high frequency stimulation to the nucleus accumbens (ventral striatum) during extinction training, indeed significantly reduced fear during extinction retrieval compared to sham stimulation controls.

## Conclusions

Collectively, these data identify potential beneficial effects of various drug treatments and DBS, including those with HDAC inhibiting or mGlu7 agonism properties, as adjuncts to facilitate the outcome of exposure-based therapies for anxiety disorders.

